# The Cerebrospinal Fluid Inflammatory Response to Preterm Birth

**DOI:** 10.3389/fphys.2018.01299

**Published:** 2018-09-12

**Authors:** James P. Boardman, Graeme Ireland, Gemma Sullivan, Rozalia Pataky, Bobbi Fleiss, Pierre Gressens, Veronique Miron

**Affiliations:** ^1^MRC Centre for Reproductive Health, The Queen’s Medical Research Institute, The University of Edinburgh, Edinburgh, United Kingdom; ^2^Centre for Clinical Brain Sciences, Chancellor’s Building, The University of Edinburgh, Edinburgh, United Kingdom; ^3^Centre for the Developing Brain, Division of Imaging Sciences and Biomedical Engineering, King’s College London, London, United Kingdom; ^4^PROTECT, INSERM, Université Paris Diderot, Sorbonne Paris Cité, Paris, France; ^5^PremUP, Paris, France

**Keywords:** preterm birth, brain injury, inflammation, cerebrospinal fluid, microglia, myelination

## Abstract

**Background:** Preterm birth is the leading risk factor for perinatal white matter injury, which can lead to motor and neuropsychiatric impairment across the life course. There is an unmet clinical need for therapeutics. White matter injury is associated with an altered inflammatory response in the brain, primarily led by microglia, and subsequent hypomyelination. However, microglia can release both damaging and trophic factors in response to injury, and a comprehensive assessment of these factors in the preterm central nervous system (CNS) has not been carried out.

**Method:** A custom antibody array was used to assess relative levels of 50 inflammation- and myelination-associated proteins in the cerebrospinal fluid (CSF) of preterm infants in comparison to term controls.

**Results:** Fifteen proteins differed between the groups: BDNF, BTC, C5a, FasL, Follistatin, IL-1β, IL-2, IL-4, IL-9, IL-17A, MIP-1α, MMP8, SPP1, TGFβ, and TNFβ (*p* < 0.05). To investigate the temporal regulation of these proteins after injury, we mined a gene expression dataset of microglia isolated from a mouse model of developmental white matter injury. Microglia in the experimental model showed dynamic temporal expression of genes encoding these proteins, with an initial and sustained pro-inflammatory response followed by a delayed anti-inflammatory response, and a continuous expression of genes predicted to inhibit healthy myelination.

**Conclusion:** Preterm CSF shows a distinct neuroinflammatory profile compared to term controls, suggestive of a complex neural environment with concurrent damaging and reparative signals. We propose that limitation of pro-inflammatory responses, which occur early after perinatal insult, may prevent expression of myelination-suppressive genes and support healthy white matter development.

## Introduction

Preterm birth is closely associated with white matter injury and life course impairments including cerebral palsy, learning difficulty, autism spectrum disorder, and psychiatric disease (Volpe, 2009; Johnson and Marlow, 2017). A characteristic feature of white matter injury is oligodendrocyte dysmaturation, which is driven in part by immune dysregulation, and results in hypomyelination (Back and Miller, 2014; Hagberg et al., 2015). Magnetic resonance imaging studies show that generalized atypical white matter tract development is often apparent in preterm infants at term-equivalent age (Batalle et al., 2017; Telford et al., 2017), which suggests that interventions to prevent injury and support normal myelination may need to be applied during the perinatal period. Therefore a priority is to better understand the immune mediators and receptors that drive preterm white matter injury in order to identify therapeutic targets that promote healthy white matter development.

Neuropathological analyses of post-mortem tissue have shown robust activation of central nervous system (CNS)-endogenous immune cells, microglia, which express pro-inflammatory markers (iNOS, TNFα, IL-1β, and IL-6) (Yoon et al., 1997; Haynes et al., 2009); and systemic inflammation due to co-morbidities of preterm birth such as chorioamnionitis and necrotizing enterocolitis, is associated with abnormal white matter on magnetic resonance imaging *in vivo* (Shah et al., 2008; Anblagan et al., 2016; Barnett et al., 2018).

Elevated levels of inflammatory proteins in blood or cerebrospinal fluid (CSF) are associated with perinatal brain injury and increased risk of adverse neurodevelopmental outcome (Yoon et al., 1996; Nelson et al., 1998; Savman et al., 1998; Bartha et al., 2004; Viscardi et al., 2004; Carlo et al., 2011; Armstrong-Wells et al., 2015; Basu et al., 2015). However, protein levels in plasma do not always correlate with those in the CSF in preterm infants with white matter injury, demonstrating that blood analyses may not reflect events in the CNS (Ellison et al., 2005; Rajkumar et al., 2018). Furthermore, a comprehensive assessment of inflammation-associated factors in preterm CSF has not been carried out. Here, we asked whether a large-scale measurement of inflammatory markers in preterm CSF, including measures of factors known to be detrimental or supportive of white matter development, could provide a broader understanding of the neuropathology of preterm brain injury.

## Materials and Methods

### Participants

We recruited two groups of neonates from the Royal Infirmary of Edinburgh between June 2014 and September 2015 who required CSF sampling, usually for the evaluation of suspected meningitis: 17 preterm neonates with mean (SD) postmenstrual age (PMA) at birth 27.14 (2.14) weeks; and 20 term infants with mean (SD) PMA at birth 39.86 (1.86) weeks. The mean (SD) PMA at CSF sampling was 29.29 (2.86) weeks for preterm infants and 40.29 (2.0) weeks for term infants. There were no significant differences in the proportion of infants with CSF contaminated by blood defined as red blood cell count >1000 cells/mm^3^ (50% versus 42%, *p* = 0.73). Methods for sampling and storage of CSF, and the clinical phenotype of participants including plasma C-Reactive Protein, full blood count, CSF total protein and glucose concentrations and CSF microscopy have been reported previously (Pataky et al., 2017). No infant in either group had meningitis; 10 out of 17 of the preterm infants and 8 out of 20 of the term infants were classified as having blood stream infection (BSI) at the time of CSF sampling, defined as either (1) blood culture grew a pathogenic bacterial species; or (2) the blood culture was negative or grew coagulase negative Staphylococcus (CoNS) *and* the infant had one or more signs of generalized infection (apnoea, temperature instability, feeding intolerance, worsening respiratory distress, or hemodynamic instability) *and* the attending neonatologist treated with IV antibiotics for ≥5 days. The difference in proportion of infants with BSI in each group was not statistically significant (*p* = 0.33).

This study was carried out in accordance with the recommendations of UK National Research Ethics Service with written informed consent from all subjects. All subjects gave written informed consent in accordance with the Declaration of Helsinki. The protocol was approved by the South East Scotland Research Ethics Committee (14/SS/044). Written parental informed consent was obtained for CSF sampling, and the study was approved by the UK National Research Ethics Service (14/SS/044).

### Custom Antibody Microarray

A custom antibody array (“G-series” from Tebu-bio/RayBiotech) against 50 human analytes was generated to detect relative levels of: activin-A (INHBA), Brain-derived neurotrophic factor (BDNF), bone morphogenetic protein (BMP)2, BMP4, BMP7, betacellulin (BTC), cluster of differentiation (CD)200, Complement 5a (C5a), C-reactive protein (CRP), Fas ligand (FasL), follistatin, furin, Galectin-3 (Gal3), granulocyte macrophage colony-stimulating factor (GM-CSF), insulin-like growth factor-1 (IGF-1), interferon-gamma (IFNγ), insulin, interleukin (IL)-1α, IL-1β, IL-2, IL-4, IL-5, IL-6, IL-8, IL-9, IL-10, IL-12p40, IL-12p70, IL-13, IL-17A, IL-17B, IL-17C, IL-17F, IL-18, monocyte chemoattractant protein-1 (MCP1), macrophage inflammatory protein 1-alpha (MIP1α), MIP1β, matrix metalloproteinase (MMP) 8, MMP-9, nerve growth factor (NGF)-β, neurotrophic factor 3 (NT3), Osteopontin (SPP1), placental growth factor (PLGF), regulated on activation, normal t cell expressed and secreted (RANTES), stem cell factor (SCF), tumor necrosis factor (TNF)-α, TNFβ, transforming growth factor-beta (TGFβ), urokinase-type plasminogen-activator (uPA), vascular endothelial growth factor-C (VEGF-C). Arrays were carried out according to the manufacturer’s instructions. Briefly, antibodies printed onto sub-arrays were dried at room temperature (RT) for 2 h, then blocked for 30 min. Fifty microliters of CSF from each case was incubated with one sub-array for 2 h at RT, then washed with gentle rocking. Sub-arrays were then incubated with biotin-conjugated sandwich antibodies for 2 h at RT, washed thoroughly, then incubated with streptavidin-Cy3. Following washes in water, slides were read at 532 nm excitation frequency.

### Antibody Array Data Analysis

Detected values of Cy3 intensity were normalized to an internal median background level on each slide. Analytes of interest were defined as those where the median value of the preterm group was outside the interquartile range of the controls. For analyses designed to generate hypotheses about group differences for gene expression studies, the distribution of values according to gestation category (preterm versus term) was investigated using independent samples Mann–Whitney *U* test, individual test *p*-values are reported, and a threshold of <0.05 was used to select proteins for microglia gene expression analysis. Analyses were performed using SPSS 21.0 (SPSS Inc., Chicago, IL, United States).

### Animal Protocol

Experimental protocols were approved by the Bichat-Robert Debre (France) ethical committee under the reference 2011-14/676-0053, and met the guidelines for the United States Public Health Service’s Policy on Humane Care and Use of Laboratory Animals (NIH, Bethesda, MD, United States). We housed the OF1 strain mice (Charles River; L’Arbresle, France) under a 12 h light-dark cycle with *ad libitum* food and water. On P1 pups were sexed, all males were kept but litters were maintained at 9–11 pups. Assessments of injury and outcomes were made only in male animals as females do not display white matter injury in response to this paradigm. The preponderance to injury in males is similar to what is observed in preterm born infants (O’Driscoll et al., 2018). Neonatal received twice a day (bid) from P1 to P4 and once on P5 a 5 μl intra-peritoneal (ip) injection of 10 μg/kg/injection recombinant mouse IL-1β in phosphate buffered saline (PBS; R&D Systems, Minneapolis, MN, United States) or PBS alone (control). IL-1β exposure, as reported previously, sets up a complex systemic inflammatory response (Favrais et al., 2011) and then a complex central neuroinflammatory response (Krishnan et al., 2017; Van Steenwinckel et al., 2018). This leads to microgliosis, oligodendrocyte maturation arrest, hypomyelination and cognitive deficits (Favrais et al., 2011; Schang et al., 2014; Krishnan et al., 2017; Van Steenwinckel et al., 2018) reminiscent of what is observed in preterm born infants (Billiards et al., 2008; Verney et al., 2012; Caldinelli et al., 2017; Spittle et al., 2017).

### Neural Tissue Dissociation and Magnetic-Activated Cell Sorting

At P1, P5, and P10, we collected brains for cell dissociation and CD11b-positive cell enrichment using a magnetic coupled antibody extraction technique (MACS), as previously described (Schang et al., 2014; Krishnan et al., 2017) and according to the manufacturer’s protocol (Miltenyi Biotec, Bergisch Gladbach, Germany). In brief, we pooled brains (*n* = 4 at P1, *n* = 3 at P5, and *n* = 2 at P10) and after removing the cerebellum and olfactory bulbs they were dissociated using the Neural Tissue Dissociation Kit. A total of six samples per group and per time point were generated with at least four independent litters per group. Using anti-CD11b MicroBeads we captured the CD11b+ cells and after elution, we centrifuged the isolated cells for 5 min at 600 g and then conserved them at -80°C. The purity of MACSed CD11B+ fraction has been validated using FACS analysis of CD11B fluorescence, and with RT-qPCR of the positive and negative cell fractions as previously described (Schang et al., 2014; Krishnan et al., 2017) and revealed the negative fraction has gene expression levels 98% lower than found in the respective primary cultures of astrocytes, neurons, and oligodendrocytes.

### Microarray Analysis

As previously published, Miltenyi Biotec (France) performed microarrays (Mouse Agilent Whole Mouse Genome Oligo Microarrays, 8 × 60K) on 6 samples per time point per group for CD11b enriched cell samples from P1, P5, and P10 mice exposed to IL-1β or PBS; a total of 24 samples (Krishnan et al., 2017). Preparation of samples for array analysis has been previously described (Husson et al., 2005; Chhor et al., 2013; Krishnan et al., 2017). The Agilent feature extraction software was used to process microarray image files. We only included signal intensities above background. Signal intensity values were background subtracted and uploaded following instructions by Miltenyi Biotec GmbH (Stefan Tomiuk) and PerkinElmer (Matt Hudson) into GeneSifter Analysis Edition v4.0^1^ for further analysis as previously described (Gustavsson et al., 2007). The pre-processed signal intensity values were median normalized, and the gene expression in neuroinflammatory and PBS controls were compared at P1, P5, and P10 using *t*-test (*p* < 0.05) with Benjamini-Hochberg multiple testing correction.

## Results

### Differential Levels of Inflammation-Associated Proteins in Pre-term vs. Term Infant Cerebrospinal Fluid

To conduct a comprehensive assessment of CNS inflammatory state in preterm infants and associate this with factors affecting myelination, we designed an antibody array assessing expression of 50 proteins with functions in regulating inflammation and myelination (**Table 1**). This approach was optimal to assess relative protein levels in neonatal CSF because it allowed high-content simultaneous screening of low volumes of fluid with high sensitivity and a broad range of detection.

**Table 1:** Median (IQR) normalized fluorescence intensity of 50 cerebrospinal fluid analytes from term control infants and preterm infants.

Analyte	Function	Control			Preterm		*p*-Value
		Median	IQR	Q1–Q3	Median	IQR	
**C5a (*Hc*)**	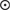	**1378.5**	**1382.5**	**680.8–2063.3**	**3592.6**	**2540.3**	**0.001^∗∗^**
CRP	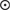	28446.4	20206.4	15120.2–35326.6	35217.1	18532.5	0.133
**GM-CSF (*Csf2*)**	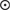	**255.6**	**91.2**	**212.2–303.5**	**330.2**	**284.2**	**0.125**
IFNγ	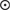 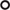	403.2	127.0	328.4–455.4	402.4	159.3	0.916
**IL-1α**	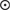	**490.5**	**81.4**	**450.6–532.0**	**581.9**	**546.13**	**0.220**
**IL-1β**	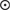 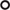	**52.1**	**73.3**	**10.0–83.3**	**108.8**	**338.8**	**0.014^∗^**
**IL-2**	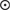	**413.1**	**77.8**	**367.7–445.5**	**520.5**	**298.0**	**0.030^∗^**
IL-6	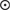 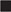	580.0	3259.5	413.8–3673.3	706.2	8133.4	0.869
IL-8	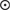	19466.8	55521.7	16192.7–71714.4	18370.4	79637.3	0.707
**IL-9**	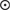 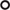	**188.8**	**76.1**	**136.9–213.1**	**242.6**	**233.23**	**0.005^∗∗^**
IL-12p40	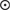	62.5	87.3	22.8–110.1	83.3	206.6	0.167
**IL-12p70**	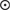	**164.3**	**80.0**	**110.1–190.0**	**220.1**	**219.8**	**0.177**
**IL-17A**	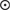 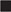	**202.1**	**45.4**	**182.5–227.9**	**296.4**	**225.6**	**0.028^∗^**
IL-17B	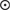	104.6	20.0	96.3–116.3	107.0	125.4	0.798
IL-17C	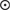	508.8	99.8	456.7–556.5	546.2	246.9	0.052
IL-17F	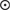	72.5	73.2	22.8–96.0	62.6	214.0	0.619
**IL-18**	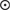	**80.9**	**49.6**	**50.0–81.0**	**101.8**	**129.0**	**0.326**
MCP-1 (Ccl2)	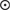	116104.0	10004.9	112697.7–122702.6	109752.5	40956.0	0.283
**MIP1α (Ccl3)**	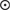	**211.5**	**282.6**	**118.0–400.6**	**1054.5**	**4168.5**	**0.015^∗^**
**MIP1β (Ccl4)**	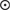	**6715.4**	**17113.7**	**5549.0–22562.7**	**26868.7**	**31793.7**	**0.133**
**PIGF**	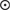	**593.2**	**375.6**	**438.0–813.6**	**948.0**	**1180.7**	**0.056**
RANTES	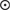	182.8	1590.6	115.7–1706.3	736.4	14673.8	0.244
**TNFα**	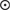 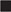 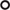	**1095.9**	**167.4**	**1036.8–1204.2**	**1242.3**	**935.8**	**0.074**
**TNFβ**	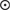	**452.6**	**68.8**	**404.4–473.3**	**504.0**	**380.4**	**0.011^∗^**
uPA	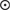	8733.4	8969.4	4470.1–13439.5	12527.5	18004.0	0.149
CD200	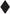	98.9	61.3	58.4–119.7	113.6	163.9	0.326
**IL-4**	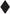	**402.7**	**80.7**	**340.7–421.4**	**430.6**	**176.6**	**0.042^∗^**
IL-5	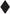	299.9	58.6	271.5–330.1	301.9	258.2	0.798
IL-10	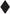 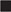	481.6	787.9	429.1–1216.9	917.7	6406.5	0.341
IL-13	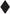	382.8	93.4	336.2–429.5	416.3	330.8	0.104
**Activin-A (INHBA)**	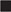 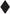	**238.2**	**62.7**	**204.9–267.6**	**271.1**	**314.6**	**0.257**
**BDNF**	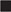	**101.6**	**18.6**	**95.3–113.9**	**145.8**	**191.5**	**0.024^∗^**
**BTC**	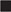	**304.7**	**49.3**	**273.3–322.6**	**354.0**	**256.7**	**0.045^∗^**
**β-NGF**	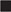	**206.4**	**169.2**	**119.1–288.2**	**339.6**	**550.56**	**0.117**
**NT-3**	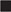	**85.7**	**55.20**	**49.9–105.1**	**106.7**	**147.0**	**0.283**
Furin	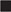 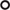	360.4	580.5	189.6–770.1	464.7	995.9	0.537
Galectin-3	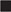	1598.1	1419.0	859.2–2278.2	1069.9	4638.26	0.892
IGF-1	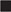	2005.4	574.4	1685.3–2259.6	2048.0	662.5	0.812
**Insulin *(Ins1)***	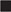	**180.6**	**70.3**	**149.0–219.3**	**253.8**	**271.8**	**0.074**
SCF (*Kitl*)	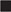	346.7	287.7	230.9–518.6	419.4	631.8	0.326
**SPP1**	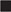 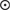	**1209.4**	**1828.0**	**766.5–2594.4**	**3064.4**	**4333.1**	**0.007^∗∗^**
**TGFβ**	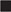 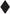	**706.2**	**122.0**	**640.7–762.7**	**919.0**	**600.0**	**0.013^∗^**
VEGF-C	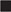	250.6	100.1	209.6–309.7	233.7	312.5	0.641
BMP2	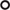	191.5	168.6	100.5–269.1	140.3	301.7	0.752
BMP4	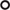	116.1	90.1	72.4–162.5	106.0	173.1	0.845
**BMP7**	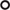	**196.5**	**211.8**	**156.2–232.0**	**246.0**	**221.5**	**0.104**
**FasL**	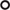	**290.1**	**159.6**	**203.1–362.7**	**967.0**	**1484.7**	**0.001^∗∗^**
**Follistatin (*Fst*)**	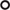	**920.6**	**286.0**	**754.3–1040.3**	**1683.0**	**1192.8**	**0.007^∗∗^**
**MMP8**	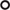 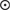	**319.7**	**187.2**	**219.5–406.7**	**416.7**	**1319.90**	**0.026^∗^**
**MMP-9**	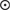	**156.4**	**126.2**	**110.8–237.0**	**244.8**	**1378.6**	**0.125**

*Bold type highlights analytes with median concentration in CSF from preterm infants that was outside the IQR of analyte concentration from controls; ^∗^p < 0.05, ^∗∗^p < 0.01. 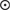
Pro-inflammatory function; 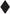 anti-inflammatory function; 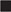
supporting oligodendrocyte lineage responses and myelination; 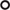 impairing oligodendrocyte lineage responses and myelination.*

We found that 28 protein levels had a median value in CSF from preterm infants that was outside the interquartile range of the controls (**Table 1**, bold type); these represented proteins with known functions in enhancing or resolving inflammation, as well impairing or supporting myelination (**Table 1**). The distribution of 15 proteins differed in preterm CSF from that of the controls (*p*-value <0.05), and 5 analytes were increased in preterm CSF at a threshold of *p* < 0.01: C5a, interleukin (IL)-9, osteopontin (SPP1), Fas ligand (FasL), and follistatin. These data demonstrate that preterm CSF has a distinct inflammatory profile compared to term controls that includes both pro-inflammatory and anti-inflammatory proteins, as well as those which can support or impair myelination.

### Mapping of Dynamic Regulation of Inflammatory and Myelination-Associated Proteins During Experimental Developmental Brain Injury

**FIGURE 1:**
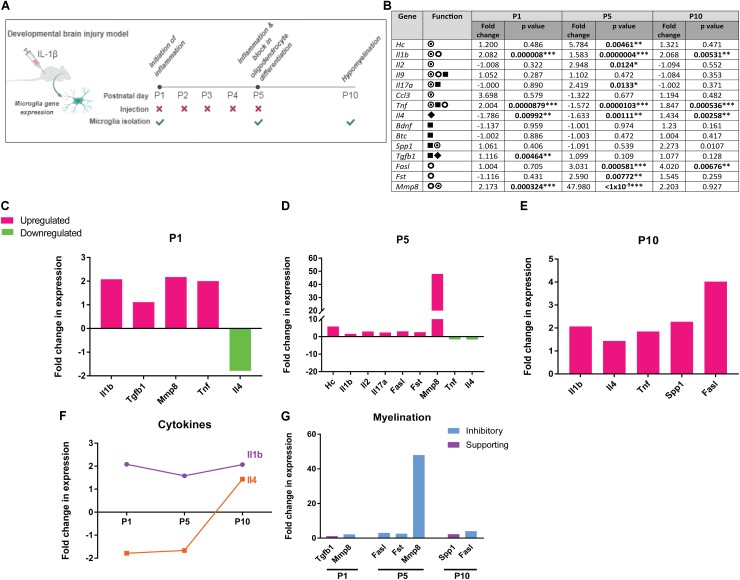
Dynamic expression of human preterm CSF signature proteins at the gene expression level in microglia following experimental brain injury. **(A)** Mouse model of developmental white matter injury. **(B)** Fold change in gene expression in microglia isolated from IL-1β mouse model of developmental white matter injury, values at postnatal (P) day 1, 5, and 10 indicated, with *p*-values ^∗^*p*<0.05, ^∗∗^*p* < 0.01, and ^∗∗∗^*p* < 0.001; 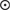 pro-inflammatory function, 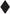 anti-inflammatory function, 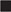 supporting oligodendrocyte lineage responses and myelination, 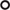 impairing oligodendrocyte lineage responses and myelination. **(C–E)** Fold change in expression of genes in microglia isolated from IL-1β injury model compared to vehicle control, at postnatal days (P) 1, 5, and 10. Significantly upregulated genes shown in magenta, significantly downregulated genes shown in green. **(F)** Dynamic regulation of *Il1b* and *Il4* over the course of injury. **(G)** Fold change in expression over vehicle control for genes associated with impairing myelination (blue) and those supporting myelination (purple).

To better understand how the proteins elevated in preterm infant CSF are regulated after injury, we mined an existing dataset in which dynamic changes in microglia gene expression were measured in an experimental model of developmental white matter injury. In this model, damage was induced by intraperitoneal injections of recombinant IL-1β (10 μg/ml) prior to the onset of myelination (twice daily from postnatal day [P]1 to P4 and one injection at P5) (**Figure 1A**). This paradigm mimics the pathophysiology of human perinatal brain injury by chronically impairing oligodendrocyte differentiation and myelination, as evidenced by immunostaining of myelin proteins, the oligodendrocyte lineage and electron microscopy (Favrais et al., 2011; Krishnan et al., 2017).

We analyzed microglia gene expression at the time of inflammation initiation (P1), during the subsequent phase when oligodendrocyte differentiation is impaired (P5), and when hypomyelination is observed (P10). Eleven of 15 proteins we found to be altered in human preterm CSF were significantly regulated at the mRNA level by microglia during the course of white matter injury (**Figure 1B**). At P1, microglia from IL-1β-treated mice showed an upregulation of pro-inflammatory genes *Il1b* and *Tnf* and concomitant downregulation of anti-inflammatory gene *Il4* (**Figure 1C**). *Tgfb1*, which supports oligodendrocyte lineage survival, proliferation, and differentiation (Dutta et al., 2014; Palazuelos et al., 2014), was upregulated simultaneously with *Mmp8*, which is associated with myelin damage (Folgueras et al., 2008; **Figure 1C**). At P5, microglia upregulated pro-inflammatory genes C5a (*Hc*), *Il1b*, *Il2*, *Il17a*, and showed a sustained downregulation of *Il4* (**Figure 1D**). Also upregulated were genes predicted to inhibit oligodendrocyte health and differentiation: *Mmp8* (Folgueras et al., 2008), *Fasl* (associated with oligodendrocyte death) (Wosik et al., 2003), and *Fst* (which would inhibit activin-A-driven oligodendrocyte differentiation) (Dillenburg et al., 2018; **Figure 1D**). At P10, *Il1b*, *Tnf*, and *Fasl* were still upregulated, however, concomitant upregulation of *Il4* and pro-myelination gene *Spp1* (Osteopontin) may indicate late attempts to resolve inflammation and counter white matter damage (**Figure 1E**). *Il1b* and *Il4*, prototypical pro- and anti-inflammatory cytokines respectively, were found to be regulated in expression throughout injury, with a sustained upregulation of *Il1b* and a delayed *Il4* response (**Figure 1F**). With regards to genes regulating myelination, although pro-survival/myelination genes *Tgfb1* and *Spp1* were slightly upregulated early and late in injury, respectively, genes whose products are predicted to impair white matter health were upregulated at all time points (**Figure 1G**). This data suggest that the proteins enriched in human preterm CSF are dynamically expressed by microglia following developing white matter insult, with an initial and sustained pro-inflammatory response followed by a delayed anti-inflammatory response, and a continuous expression of genes predicted to inhibit healthy myelination.

## Discussion

In this study, we identified a distinct inflammatory signature in preterm CSF relative to term controls. A comprehensive and sensitive measure of CSF protein levels by antibody array identified 15 factors which were relatively increased in preterm samples, and which have been previously associated with regulating inflammation and myelination. This revealed a complex preterm neural environment, with concurrent pro- and anti-inflammatory responses, and pro- and anti-myelination factors. Data-mining of microglia transcriptomes in the context of experimental perinatal brain injury revealed that microglia can express the majority of these factors following insult, and they are dynamically regulated over time, mirroring the complexity of inflammatory and myelination-regulating factors measured in the human samples. Although some of these genes are expressed by microglia in healthy developing brain (*Il1b, Ccl3, Tnf, Spp1*, and *Tgfb1*) (Zhang et al., 2014), the rapid increase of pro-inflammatory gene expression in microglia in this experimental model shortly after the first injection of IL-1β, concomitant with sustained expression of genes predicted to impair myelination, highlight the importance of early intervention to limit damage to the developing white matter.

Five proteins were increased in preterm CSF vs. controls at the threshold *p* < 0.01: C5a, IL-9, SPP1, FasL, and Follistatin. C5a is a component of the complement cascade which we previously showed to be increased in human preterm CSF by enzyme-linked immunoabsorbant assay (Pataky et al., 2017), validating our novel approach of using antibody array to identify differentially expressed proteins in CSF samples. Although C5a has been associated with normal brain development (Benard et al., 2008) and neuroprotection (Biggins et al., 2017), it may have damaging functions as inhibition of its receptor C5aR attenuates excitotoxic perinatal brain injury (Pedroni et al., 2013) and C5a is increased in the CSF of children with demyelinating disease (Horellou et al., 2015). In addition, we identified IL-9 as a novel preterm birth-associated CNS cytokine in humans. Although we found it is not regulated by microglia in the IL-1β injury model, it may be expressed by other cell types such as Th9 lymphocytes. In experimental models it has roles in mast cell activation and excitotoxicity (Patkai et al., 2001), neonatal cortical neuronal apoptosis (Fontaine et al., 2008), autoimmune demyelination (Li et al., 2011), and regulation of astrocyte chemokine production (Ding et al., 2015); our data support a role for IL9 in the human inflammatory response to preterm birth. In addition, its expression can be driven by TGFβ (Beriou et al., 2010), which when overexpressed by microglia is associated with hypomyelination (Nobuta et al., 2012). IL-9 may also have direct actions on oligodendrocyte lineage cells, as these express the IL-9 receptor and IL-9 treatment inhibits their differentiation *in vitro*, although, notably, it can encourage differentiation if co-supplied with IFN-γ (Ding et al., 2015).

The remaining three highly enriched proteins have been implicated in regulating the oligodendrocyte lineage and myelination. Osteopontin (SPP1) in particular has been suggested as a blood biomarker for neonatal encephalopathy (Graham et al., 2018) and it is highly induced by hypoxic injury, where a protective role is suggested by decreased oligodendrogenesis in a knockout mouse subjected to hypoxic-ischemic injury (van Velthoven et al., 2011). This beneficial role may be dependent on mode of neural injury or age, as in adult mice SPP1 exacerbates autoimmune-mediated demyelination and is not required for regeneration of myelin on previously myelinated axons (Zhao et al., 2008). Nonetheless, it can directly increase myelin protein expression and myelination *in vitro* (Selvaraju et al., 2004). Another protein we detected in preterm CSF which may directly modulate myelination is follistatin, which sequesters activin-A to prevent its binding to activin receptors. This would be predicted to impair myelination, as we have recently shown these receptors to be required for oligodendrocyte differentiation and myelin maturation in healthy white matter development and following injury (Dillenburg et al., 2018). Lastly, the increase in Fas ligand in preterm CSF may indicate direct targeting of oligodendrocytes, as it has been associated with induction of oligodendrocyte death in a variety of neurological disorders (Austin and Fehlings, 2008).

Our study has some limitations. Although no infant in the study group had meningitis, a proportion of preterm and term infants had BSI at the time of CSF sampling. Therefore, it is possible that systemic inflammation contributed to observed alterations in the CSF inflammatory profile, although this potential confounding effect is likely to be balanced across the groups.

Our study was not designed to investigate the effect of astrocyte-mediated cytokine production, which could contribute to neuroinflammation as has been suggested by some experimental perinatal white matter injury models (Nobuta et al., 2012; Shiow et al., 2017). In future work, investigating gene expression in other cell types including astrocytes and neurones may be informative.

Altogether, these findings identify a CSF signature in response to preterm birth, which reflects a complex environment that can both drive injury and support myelination. The pathological outcome of preterm birth may thus reflect a balance between damaging and reparative factors, which implies that effective therapies may need to operate on multiple targets. We propose that early therapeutic intervention could pre-empt the robust pro-inflammatory response and boost pro-repair mechanisms to support healthy myelination.

## Data Availability

All human CSF data generated or analyzed during this study are included in this published article. The microglia gene expression datasets analyzed during the current study are available from the corresponding author on reasonable request.

## Author Contributions

JB co-designed the study, analyzed the patient data, and contributed to writing the manuscript. GI and GS analyzed the patient samples. BF and PG co-designed the study, carried out the animal experiments, and generated the microglial gene expression dataset. RP collected the human CSF samples. VM co-designed the study, analyzed the microglia gene expression dataset, and co-wrote the manuscript. All authors read and approved the final manuscript.

## Conflict of Interest Statement

The authors declare that the research was conducted in the absence of any commercial or financial relationships that could be construed as a potential conflict of interest.

## Footnotes

1 http://jhu.genesifter.net/login
